# Novel Therapeutic Strategies Applied to *Pseudomonas aeruginosa* Infections in Cystic Fibrosis

**DOI:** 10.3390/ma12244093

**Published:** 2019-12-07

**Authors:** Michael E. Chirgwin, Margaret R. Dedloff, Alina Maria Holban, Monica C. Gestal

**Affiliations:** 1 Department of Chemical Engineering, Clarkson University, Potsdam, NY 13699, USA; chirgwme@clarkson.edu; 2 Department of Biology, Clarkson University, Potsdam, NY 13699, USA; dedlofmr@clarkson.edu; 3 Department of Microbiology, Faculty of Biology, University of Bucharest, 030018 Bucharest, Romania; alina_m_h@yahoo.com; 4 Research Institute of the University of Bucharest (ICUB), 050107 Bucharest, Romania; 5 Department of Science and Engineering of Oxide Materials and Nanomaterials, Faculty of Applied Chemistry and Materials Science, University Politechnica of Bucharest, 1-7 Polizu Street, 011061 Bucharest, Romania; 6 Department of Infectious Diseases, College of Veterinary Medicine, University of Georgia, Athens, GA 30602, USA

**Keywords:** liposomes, nanoparticles, *Pseudomonas aeruginosa*, cystic fibrosis, bacteriophages

## Abstract

Cystic fibrosis (CF) is one of the most prevalent genetic diseases and a total of 1700 different genetic mutations can cause this condition. Patients that suffer this disease have a thickening of the mucus, creating an environment that promotes bacterial infections. *Pseudomonas aeruginosa* is a ubiquitous bacterium, which is frequently found in the lungs of CF patients. *P. aeruginosa* is known for its high level of antibiotic resistance as well as its high rate of mutation that allows it to rapidly evolve and adapt to a multitude of conditions. When a CF lung is infected with *P. aeruginosa*, the decay of the patient is accelerated, but there is little that can be done apart from controlling the infection with antibiotics. Novel strategies to control *P. aeruginosa* infection are imperative, and nanotechnology provides novel approaches to drug delivery that are more efficient than classic antibiotic treatments. These drug delivery systems are offering new prospects, especially for these patients with special mucus conditions and bacterial characteristics that limit antibiotic use.

## 1. Introduction

Cystic fibrosis (CF) is an inherited disease caused by a mutation that affects the cystic fibrosis transmembrane regulator (CFTR), leading to the generation of a dysfunctional protein impeding chloride from reaching the cell surface [1]. This causes the body to produce and build up thick mucus that accumulates in the respiratory, digestive, and reproductive systems and first afflicts only small airways, but further progresses to affect all of them. The special characteristics of the composition and consistency of this mucus enhances susceptibility to lung infections by trapping bacteria that can usually be cleared through the usual clearance mechanisms [2]. Currently there is no cure for CF, but there are stringent antibiotic regiments to improve life expectancy by about 40 years [3].

One opportunistic bacteria commonly isolated from patients with CF is *Pseudomonas aeruginosa*, which is a gram-negative bacteria that is usually found in soil and water [4]. Infections caused by this pathogen often occur in patients hospitalized or those with weakened immune systems. It is responsible for a great number of multi-drug resistant nosocomial infections [3]. *P. aeruginosa* infects about 51,000 patients in the US each year, with about 6700 being multidrug-resistant and it was responsible for 2700 deaths in 2017 according to the Centers for Disease Control and Prevention [5]. In patients with CF, 48% of the patients are infected with *P. aeruginosa* and infection occurs with the median age of 6 at first infection, provoking a decline in their lung function that will be accelerated [6]. Importantly, *P. aeruginosa* possesses different mechanisms to manipulate or evade host immune response, including a mucoid exopolysaccharide, which is known as alginate. Remarkably, alginate slime promotes biofilm formation as it anchors the cells to the environment, which is able to protect against immune defenses. This ability of *P. aeruginosa* to manipulate host immunity results in a worsening of the disease in this population associated to formation of biofilm and other phenotypes.

Biofilm formation of *P. aeruginosa* is a major concern for antibiotic use and resistance. In in vitro models, the tolerance of the biofilm to antibiotics is mainly associated with the mode of growth. This can be seen with planktonic cultures, which are typically more susceptible to antimicrobials [7]. Mono and polymicrobial biofilms are able to avoid immune cell phagocytosis, while triggering a persistent, low intensity inflammatory response. Planktonic bacteria, on the other hand, invoke a powerful systemic response, which allows for a more effective use of antibiotics [8]. In infections with more than one microorganism, the different species can exchange nutrients to sustain one another’s proliferation [9]. Importantly, biofilms are able to restrict the penetration of antimicrobials because of the formation of a matrix. This matrix cannot inhibit diffusion but it can restrict the penetration due to an antimicrobial’s nature of binding to compounds inside the biofilm or the bacterial membranes. The extracellular polysaccharide matrix (EPS) has been demonstrated to be the main reason biofilms are able to resist phagocytosis by preventing opsonins on the bacterial wall and by blocking penetration [10]. *P. aeruginosa* biofilms have shown that metabolic activity of the bacteria is higher in the outer portion of the biofilm than the inner portion. This can be explained by the limited oxygen nutrient penetration through biofilm because of bacterial consumption [7]. Persister cells are also contained within the *P. aeruginosa* biofilm in small amounts, typically less than 0.1%. These cells are slow dividing or are non-dividing bacteria that show diminished susceptibility [11]. Similarly, *in vivo* models show similar effects as in vitro models and some additional factors need to be considered. Sputum in patients with CF and chronic infections of *P. aeruginosa* has polymorphonuclear leukocytes (PMN), which accumulate at the site of biofilm formation and consume oxygen to create anaerobic conditions and restrict bacterial growth. In this low oxygen environment, reactive oxygen species (ROS) dependent effects of fluoroquinolones, beta-lactams, and aminoglycosides are greatly affected. This also inhibits the mechanism enacted by aminoglycosides, which depend on oxygen to be transported across the membrane. When a low dose of antibiotic is administered, bacteria are not killed and instead increase the resistance due to selection and mutagenesis [7]. Therefore, high antibiotic dose regiments are often used to fend off infections.

The current treatment for *P. aeruginosa* infections in CF patients involves inhaled antibiotic treatments to directly target the lungs, however, these formulations are rapidly absorbed and cleared from lungs leaving bacteria free to growth. To ensure therapeutic dosage at the site of infection, multiple administrations of the drug are required each day, increasing not only the cost of treatment but also producing a burden for the patients that have to strictly administer the dosages, affecting their personal life and schedules [12]. Current drugs being used are aminoglycosides, carbapenems, cephalosporins (third and fourth generation), fluoroquinolones, monobactam, extended-spectrum β-lactamases, and polymyxin B/Colistin, the last being highly nephrotoxic for the patients [13]. Currently, researchers are developing novel methods that combine the classical inhaled forms of these products with the use of liposomes, solid lipid nanoparticles, polymeric nanoparticles, and bacteriophages to provide sustained drug release for an increase in patient compliance [14]. In this review we will compile the new data regarding novel methods and approaches to treat *P. aeruginosa* infections in this population that is highly susceptible.

## 2. Inorganic Nanoparticles

Nanoparticles (NPs) are nanoscale materials with a great impact on the biomedical field. Such materials were clustered in inorganic and organic nanoparticles, depending on their composition. Both organic and inorganic nanoparticles proved intrinsic antimicrobial properties and they are widely utilized as vectors for the delivery of natural and synthetic antimicrobials [15]. Inorganic NPs, such as silver NPs proved their antimicrobial efficiency against CF recovered multidrug resistant pathogens, such as *P. aeruginosa*, *Burkholderia cepacia*, *Stenotrophomonas maltophilia*, and *S. aureus* strains [16]. Silver NPs (AgNPs) proved highly bactericidal effect on the drug-resistant or multidrug-resistant *P. aeruginosa* with the minimum inhibitory concentration (MIC) range of 1.406–5.625 µg/mL and the minimum bactericidal concentration (MBC) range of 2.813–5.625 µg/mL. The main antimicrobial mechanism involves the disequilibrium of oxidation and antioxidation processes and the failure to eliminate the excessive reactive oxygen species (ROS). Studies demonstrated that AgNPs could enter the multidrug-resistant bacteria and impair their morphology and structure. Moreover, in the AgNP-treated bacteria, the levels of Superoxide dismutase, catalase, and peroxidase such as alkyl hydroperoxide reductase and organic hydroperoxide resistance protein, were obviously high, as well as the significant upregulation of low oxygen regulatory oxidases, including cbb3-type cytochrome c oxidase subunit P2, N2, and O2 [17]. Recent studies have demonstrated that nanoparticles could enhance the antimicrobial properties of antibiotics against CF *P. aeruginosa* strains. Chen and coworkers tested a combination of minocycline and silver dual-loaded polyphosphoester-based nanoparticles for the treatment of resistant *P. aeruginosa* strains. They used 4-epi-minocycline, a metabolite of minocycline, identified as an active antimicrobial against *P. aeruginosa* using a high-throughput screen. The antimicrobial activities of 4-epi-minocycline, minocycline, and silver acetate against clinical isolates of *P. aeruginosa* obtained from CF patients were evaluated in vitro. The results demonstrated that both silver and minocycline are potent antimicrobials alone and that the combination allows for a reduced dosage, while promoting a robust antimicrobial effect. Furthermore, the proposed synergistic silver/minocycline combination can be co-loaded into nanoparticles as a next generation approaches to combat the threats presented by multidrug resistant (MDR) pathogens [18]. Research conducted by Grumezescu et al. uses biocompatible iron oxide NPs to increase the efficacy of amoxicillin (AMO) delivery throughout the body to gram negative and gram positive bacteria. The synthesized 10 nm Fe_3_O_4_@AMO NPs were found to have low toxicity in an in vivo murine model. These NPs were found to localize in the lungs, kidneys, and spleen while being totally absent from the brain and liver. Effects were observed on both gram negative and gram positive bacteria with a reduced minimum inhibitory concentration (MIC) of amoxicillin [19].

Magnetite NPs (MNPs) cross-linked with chitosan and coated with aminoglycoside antibiotics (kanamycin and neomycin) were also used to stop the growth of *P. aeruginosa* [20]. The incorporation of the NPs with these antibiotics clearly enhanced the antimicrobial activity of the last due to the higher surface area to volume ratio and to the controlled release of the aminoglycosides. MNPs functionalized with eugenol had also similar anti-adherence activity against *P. aeruginosa* strains [21], making them ideal candidates for developing new anti-bacterial materials. Effective against *P. aeruginosa* are also the MNPs coated with chitosan-carboxymethylcellulose and incorporated with antibiotics [22]. Nanocomposites consisting of biogenic magnetite, silver NPs and chitosan exhibited MIC values between 14 and 50 mg/L against *P. aeruginosa*, lower than the plain drugs [23]. MNPs stabilized with thioglycerol showed also a good inhibition against *P. aeruginosa* with a MIC value of 0.047 mg/mL.

Despite their proved efficiency against *P. aeruginosa* strains, the antimicrobial mechanisms of inorganic NPs and their long-term effect on the host are still under investigation.

## 3. Liposomes

Liposomes are phospholipid vesicles with hydrophobic bilayers alternating with aqueous compartments [24]. Liposomes are ideal for drug delivery due to their structure of the lipid bilayer mimicking other cells and thus increasing interactions with the host immune system. Liposomes are very stable when nebulized and thus can be easily administered directly to the lungs in combination with antimicrobials and other drugs [25]. Currently, liposomes are widely utilized and investigated in the cystic fibrosis therapy. For example, liposomes containing beclomethasone (glucocorticoid steroid) and formoterol (β2-selective receptor agonist) showed that beclomethasone maintained its long-lasting effect while formoterol enhanced lung function and peripheral lung deposition without a significant effect on the mucociliary escalator [26]. The targeted delivery of antimicrobials by inhalation has also received much interest in the past years. Indeed, some liposomal formulations of antibiotics for aerosolized delivery to the lungs are currently in phase II clinical trials [27]. In particular, the liposomal amikacin (Bristol-Myers Squibb, New York, NY, USA) for inhalation therapy showed sustained improvement in lung function and significant reduction of bacterial density in CF patients with chronic *P. aeruginosa* lung infections [28]. Similarly, the antifungal drug amphotericin B formulated in liposomes showed reduced side-effects and increased pulmonary deposition and retention in animal models of aspergillosis compared to non-liposomal amphotericin B [29], and was well tolerated in immunocompromised patients [30]. In another approach by Vij et al., a polymer-based nano-delivery system showed delivery of a proteasome inhibitor to the lungs of mice in a controlled and sustained manner, which could rescue *P. aeruginosa*-lipopolysaccharide induced CF lung disease [31].

**Figure 1:**
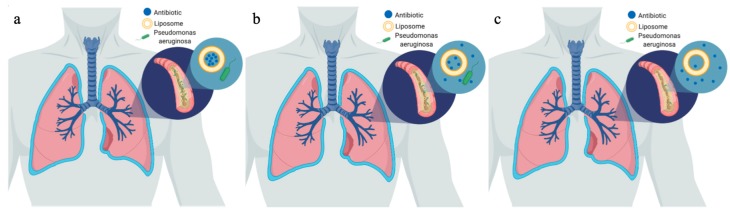
Liposomal controlled release diagram. (**a**) shows the liposomes after administration to the body in the airways of a patient with cystic fibrosis (CF) and pseudomonas infection. (**b**) shows the release of antibiotic after 10.5 h. (**c**) shows the release of antibiotic after 21 h.

Ciprofloxacin is a broad-spectrum antibiotic feasible for treatment of both gram-negative and gram-positive bacteria. Liposomes containing ciprofloxacin have demonstrated the ability to provide a controlled and sustained release of 1.5% of the drug crossing the epithelium longer than four hours. This is contrasted with greater than 33% of the free drug formulation crossing in the same time. The slow release is due to the slow decomposition or modifications to the liposome membrane, allowing the drug to gradually emanate from the vesicle or gentle depletion of the transmembrane ion gradient, resulting in more uncharged drug transported across the bilayer. When comparing the liposomal and free drug formulations against *P. aeruginosa* and *S. aureus*, the results indicated a higher activity against gram-negative *P. aeruginosa*. It is worth to note that gram-negative bacteria have a negatively charged lipopolysaccharide (LPS) layer, which causes a fusion-interaction of the negative phospholipids in the liposomes. On the other hand, the phospholipids are not able to form strong interactions with the thick peptidoglycan layer of gram-positive bacteria, and thus this delivery is less effective for *S. aureus* [12]. In a similar study conducted by Bruinenberg et al., once a day inhalation of liposomal ciprofloxacin was achieved with a half-life of 10.5 h and 99% encapsulation [32] (Figure 1).

Aminoglycosides are highly positively charged, enabling them to become strongly bound to the negatively charged polymers in infected mucus such as DNA, alginate, and mucins [33]. Amikacin has also been successfully encapsulated in highly stabile liposomes formed by dipalmitoyl phosphatidylcholine and cholesterol, which are naturally found in lung surfactant. These liposomes can penetrate biofilm and be released in a controlled manner via diffusion at the site of infection [33]. This is a characteristic that has great impact in the treatment of cystic fibrosis, as most patients present high levels of biofilm formation in their lungs. Zwitterionic lipids have similarly enhanced penetration and remarkably, these particles are able to organize in a biofilm-like structure such as that in *P. aeruginosa*. It was found that about 10^10^ bacteria released enough rhamnolipid to release amikacin from about 1000 liposomes. Tobramycin has also been incorporated into liposomes to treat *P. aeruginosa*. Liposome-encapsulated tobramycin achieved high levels of 91.08 micrograms/pair of lungs after 15 min and remained constant for the next 16 h, compared to the free tobramycin, which had concentrations 4.5 times lower than encapsulated tobramycin after 15 min and was fully cleared within 3 h in uninfected rats. In the infected rats, encapsulated tobramycin revealed higher concentrations of antibiotic after 15 min than the free form, but was cleared after 1 h compared to the constant release of tobramycin from the liposomes for 16 h [34]. This has shown that the availability time within the lung and sustained release was greatly improved by using liposomes as the delivery method.

Polymyxin B, a polycationic peptide antibiotic, is highly effective at treating infections of gram-negative bacteria but unfortunately is not commonly used due to its elevated cytotoxicity. This antibiotic works by interacting with acidic phospholipids and lipopolysaccharide of membranes, disrupting the structure of the outer cell wall. However, due to the high levels of resistance in some bacteria, this antibiotic is the only available treatment for the patient. Liposomes effectively prolong the interaction of polymyxin B and the site of infection, while decreasing toxicity. Free polymyxin B may cause nephrotoxicity or neuromuscular blockades, but when encapsulated and released from liposomes, there were no measurable quantities of polymyxin B in kidneys or serum of rats. The LPS of bacteria has also been shown to accumulate neutrophils and injure endothelial cells, which leads to leakage across the microvascular basement membrane, but polymyxin B is able to alleviate this injury and edema by neutralizing the LPS [24].

Liposomes have been used to carry and prolong the release of tobramycin in a study done with Sprague-Dawley rats, where the colony forming units (CFU) count of 0–3 was significantly lower than the minimal 30 CFU counts. Liposomes created with dipalmitoyl phosphatidylcholine (DPPC) and dimyristoyl phosphatidylglycerol (DMPG) were more effective than liposomes created with distearoyl phosphatidylcholine (DSPC) and dimyristoyl phosphatidylcholine (DMPC), which did not have any greater effect than the control group of rats treated with phosphate buffered saline (PBS). Excitingly, only two doses of 240 micrograms of tobramycin in DPPC-DMPG were sufficient for the treatment instead of the typical three doses of 600 microgram of free tobramycin [35].

Liposomes coated with polyethylene glycol with ciprofloxacin were tested in rat models of acute and chronic *P. aeruginosa* infections. Rats infected with *P. aeruginosa* experienced 89% mortality within 24–48 h of inoculation. When similar rats were treated with a single dose of 40 mg/kg pegylated liposomal ciprofloxacin on the first day and nonliposomal ciprofloxacin at 40 mg/kg per day after initial dose for seven days, survival increased to 80%. A survival of 100%was achieved when pegylated liposomal ciprofloxacin was administered at 160 mg/kg twice daily [36].

Overall, liposomes are promising for drug delivery due to their ability to increase bioavailability and have targeted and sustained release.

## 4. Solid Lipid Nanoparticles

Solid lipid nanoparticles (SLN) are more stable than liposomes and do not have a bilayer structure, but instead they have a matrix structure that is able to hold either lipophilic or hydrophilic drugs. Some of the major drawbacks include low drug loading and unpredictable drug release, but they are less cytotoxic and have the potential for high drug payload [37].

Quorum sensing (QS) is the mechanism that several bacteria used to coordinate their behavior using small signal diffusible molecules. PqsR, one of the main regulatory proteins of the QS in *P. aeruginosa*, that binds to the receptor that controls pyocyanin, elastase B, and hydrogen cyanide, other important *P. aeruginosa* virulence factors and I has been the target for some of these therapies. A promising therapy to treat *P. aeruginosa* infections utilizes a quorum sensing inhibitor (QSI) that disrupts bacterial communication blocking the ability to coordinate behavior to form a biofilm [38]. Encapsulation efficiency of the QSI was 60%–95% due to polymorphic changes of the lipid to less ordered structures, and several different compounds have been tested in order to improve encapsulation efficacy. Some of these compounds include precirol, which had the highest encapsulation efficiency, or tristearin, that although was most stable, revealed lower encapsulation efficiency. However, when tristearin was coupled with poloxamer as the surfactant, the encapsulation efficiency significantly increased. These particles have potential for reaching deep lungs, 80% were below 5 micrometers in size. Particles that are greater than 5 micrometers are deposited in upper airways and are unable to travel to the deep lungs [38]. Due to the size variability of this study’s NPs, infections can be treated with whichever NP is more effective for the region of the lungs.

Amikacin is able to inhibit protein synthesis by binding to 30S ribosomal subunit and is one of the most commonly used treatments for *P. aeruginosa* infections. Ghaffari et al. have been able to successfully load solid lipid nanoparticles with amikacin and the results demonstrated that these conjugates were more effective than free amikacin. This could be due to the easier diffusion of SLNs into the cellular membrane of *P. aeruginosa* cells due to the similarity of the cholesterol to the cell wall of the bacteria [37]. SLNs have also been shown to have decreased toxicity to the body, and the concentration of amikacin in the kidneys was lower with the combined particles than that of free amikacin [39].

A study of tobramycin and solid lipid nanoparticles was conducted for their pharmacokinetic effects and tissue distribution. Only 2.5% of tobramycin was encapsulated by the SLNs using warm oil-in-water microemulsions in a cold aqueous medium. Usually tobramycin is not absorbed by the small intestine, but when incorporated with SLNs, it could be absorbed. When administered, the free tobramycin solution reached a peak concentration of 10 micrograms/mL, whereas the SLN-tobramycin reached 37 micrograms/mL after 60 min and 10 micrograms/mL after 24 h [40]. After duodenal administration, there was a higher concentration in lungs for the SLN-tobramycin than free tobramycin after 24 h. This is because the SLNs are able to deceive the P-glycoprotein, which lowers the bioavailability of free tobramycin, by masking the tobramycin and getting across the blood brain barrier [41].

Biodistribution studies of sodium colistimethate conjugated SLNs in a murine model revealed that after nebulization, the drug was found in the snout and oropharyngeal cavity, and further spread to the respiratory and digestive tracks after 2.5 h, where it remained for 48 h. There was no drug detected in the liver, kidney, or spleen at any point when using this delivery system suggesting that the drug is safe for human usage [42].

Solid lipid nanoparticles show the potential as an effective drug delivery method for the treatment of *P. aeruginosa*. They have been shown to have sustained drug release and low cytotoxicity, even though drug loading is low. Although mouse studies demonstrated the efficacy of antibiotic-SLPs conjugates, clinical studies will need to be performed to better understand the immunomodulatory properties of these compounds.

## 5. Polymeric Nanoparticles

Dendrimers are a type of polymer that displays high monodispersity and molecular dimensions similar to proteins. These types of molecules allow for conjugation of soluble enhancers, drugs, targeting ligands, and proteins. The half-life of the dendrimer can be increased with higher molecular weights and that dendrimer stability and retention in the lung increased with increasing chain length of polyethylene glycol (PEG), importantly, longer chain length of PEG was shown to conjugate to glucagon-like peptide (GLP-1), which increased the stability of GLP-2 and produced a greater therapeutic response of intratracheal administration in rats [43].

Poly(lactic-co-glycolic acid) (PLGA) is a polymer commonly used for drug encapsulated nanoparticles (NP) due to its low cytotoxicity. The two common types of PLGA are Resomer^®^ RG 502 H and RG 756, which differ in lactic/glycolic acid ratio and terminal group. Chitosan-alginate PLGA nanoparticles presented increased absorbance at 650 nm after addition to the mucin-coated medium due to the interactions of positively charged chitosan and the negatively charged mucin. Importantly, the chitosan-alginate RG 502 H NPs displayed a similar burst to PVA-alginate NPs, both having a higher burst compared to chitosan-alginate RG 756 NPs. These NPs can be frozen when conjugated with lactose microparticles for enhanced administration. Chitosan NPs (positively charged chitosan-alginate PLGA) accumulate in the upper airways, while the PVA-alginate NPs (negatively charged) can reach the deep lung [44].

Ciprofloxacin has revealed 79% encapsulation efficiency and drug release in water can last at least 8 h with no burst effect. In in vivo solutions, which were phosphate buffered saline with/without Tween 80 and lung fluid, 80% was released in 8 h and 90.5% released after 14 days. The drug release kinetics is driven by diffusion and follows Fick’s diffusion laws, which state that the flux of material moves from high concentrations to low concentrations. Moreover, when ciprofloxacin is coupled with the NPs, there is an increase in antibacterial activity, which could be due to steric protection of the functional group on position 7 of ciprofloxacin [45].

Further advancements focus in controlled drug release using PLGA NPs. In combination with ciprofloxacin, drug was released from PLGA NPs for 30 days, with a burst release for the first 2 days. When magnetic NPs were incorporated into PLGA micro and nanoparticles, release of the drug was controlled using an oscillating magnetic field [46]. This would be beneficial by releasing the drug by turning on a magnetic field after the NPs reach the site of infection.

An alternative mechanism to induce drug release is using rhamnolipid, levofloxacin, and ofloxacin, which have high solubility and high permeability, and can be readily released without any triggering agent due to their water solubility. When rhamnolipid or Triton X-100 was added, 60% of the drug was released in five minutes from both the hybrid NP and lipid vesicles. Ciprofloxacin, with low solubility and low permeability, had low loading and encapsulation efficiency at 0.5% and 5%, allowing for 15% released after 24 h, importantly, triggering drug release had no effect in the overall amount of drug released [47]. From this data, it can be concluded that triggering release can be effective for some types of drugs, but not for types I or IV.

Gentamicin, commonly used to treat *P. aeruginosa* infections, however, there is a great concern regarding its short half-life and low bioavailability, both of which can be augmented by conjugating gentamicin with PLGA NPs. Gentamicin is a hydrophilic molecule, which makes harder the encapsulation. One way to improve it is by increasing the pH from the typical 5 to physiological pH of 7.4 to increase the encapsulation threefold. This is because gentamicin has four amino moieties, so increasing the pH favors the deprotonation of the groups, which decreases the hydrophilicity. Previous studies have demonstrated that 50% of gentamicin was released in 24 h, and continued to release drug for at least 16 days, showing that the half-life has greatly improved. Further studies with *P. aeruginosa* biofilm revealed that the lungs of patients with CF could be treated using exchange dialysis by refreshing media every 60 min. After 36 h with mimicked CF lungs, the encapsulated drug showed greater antibiotic activity than free gentamicin, showing the sustained release of the drug from the NPs. It was also proven to have sustained release in CD 1 mice in which after 48 h, the weight of saline and free gentamicin treated mice significantly decreased compared to no reduction seen in mice treated with gentamicin NPs demonstrating that the drug release was stickily controlled by the conjugation with NPs [48].

The CF lung is mostly characterized for a neutrophilic type of inflammation, to avoid damage, treatment that targets the inflammation is common and the use of antibiotics combined with corticosteroids is usual. PEG-PLGA NPs provide sustained drug release up to 11 days in murine lungs and control NFKB mediated neutrophil levels and inflammation. Importantly, when compared with free drug, the latter is not able to control the inflammation and neutrophil levels, which is indicative of low bioavailability. The PEG-PLGA NPs were able to selectively inhibit proteasome mediated homeostatic processes in the lung epithelia using PS-341 (pyrazycarbonyl-Phe-Leuboronate) [31].

An alternative approach to deliver antibiotics is through the use of deoxyribonuclease I (DNase) with chitosan-alginate NPs in combination with tobramycin. DNase reduces the viscoelasticity of the mucus by cleaving the DNA, which increases drug penetration into the mucus allowing them to release 45% in the first 90 min and 80% after 48 h. Using the *Galleria mellonella* model, it was proven that there was no impact of the NP tobramycin compared to the free tobramycin, unless the NP was given 96 h before infecting the organism with *P. aeruginosa*. The survival rate increased from 40% to 80% with the used of combined NP-tobramycin [49].

Polymeric NPs can also be used to release cationic antimicrobial peptides (CAMPs). CAMPs are able to target the bacterial cell membrane by attaching to the anionic LPS of gram-negative bacteria, leading to LPS disassembly and increasing permeability of the cell, causing cell death. Other anionic cells can inhibit this LPS attraction, such as mucin or alginate. Colistin has poor bioavailability, thus requiring twice a day nebulization, however, chitosan coated PLGA NPs provided a more rapid release of 50% of colistin in 6 h compared to PVA coated PLGA NPs releasing 5%. The chitosan PLGA NPs easily penetrate artificial mucus layer in comparison with the PVA PLGA NPs, with 70% of chitosan NPs passing through in 6 h compared to 40% PVA NPs. After the NPs were embedded into lactose loaded nano embedded microparticles (NEM) for aerosolization, both chitosan and PVA removed 50% of biomass in first 24 h, which was worse than the free drug removal of 90% of biomass. The free drug, however, was not effective after 72 h, but both the NEMs still provided 30% biomass removal [50].

Along with their antimicrobial effect, polymeric nanoparticles were proved to inhibit some virulence features of *P. aeruginosa* strains, such as attachment and biofilm formation [51]. Since biofilm formation is the most important parameter in establishing persistent infections, such properties recommend polymeric nanoparticles as efficient structures for anti-pathogenic strategies in *P. aeruginosa* lung infections.

## 6. Bacteriophages

Bacteriophages are viruses that have the ability to infect and kill bacteria. They have been used to treat bacterial infections for many years but have fallen out of favor since the discovery of antibiotics [52]. In recent years, due to the increase in antibiotic resistance, there have been advances that utilize these unique microbes for the delivery of drugs. Specifically, there have been significant breakthroughs in using bacteriophages as a drug delivery system to treat *P. aeruginosa* infection in cystic fibrosis patients.

Several studies have investigated the use of novel dry powder inhalers containing bacteriophages for their ability to treat *Pseudomonas* spp. infection. Golshani et al. tested a dry powder inhaler containing two bacteriophages in an idealized metal mouth-throat replica. Nevertheless, because this study was only performed in vitro, it was unable to show the efficacy of the powder inhaled bacteriophages against *P. aeruginosa*, but their results show that this method was able to deliver controlled doses of the bacteriophages, suggesting that this drug delivery system may have promise in treating infection in cystic fibrosis testing [53]. The use of process phages specific for *P. aeruginosa* into dry respirable forms by spray drying enable bacteriophages to be processed and delivery in the lungs presents a great opportunity for treatment of this type of infections [54]. Additionally, Sahota et al. examined the ability of aerosolized phages to kill isolates of *P. aeruginosa*, demonstrating that phages were able to kill a high percentage of strains tested and that high titers of phages can be nebulized in vitro [55]. Using inhalers as a delivery system for bacteriophages has been shown to have potential as a future drug delivery system for treatment of *Pseudomonas* infection, but more in vivo research is necessary to fully investigate the potential of this drug delivery system.

Using a nonlytic bacteriophage to deliver DNA encoding bactericidal proteins to bacteria, the phage deliver proteins causing the bacteria to express genes that finally lead to cell death. When tested in mice, those treated with the phage carrying the bactericidal proteins experienced a significant decrease in circulating bacteria [56]. While this was tested using *Escherichia coli,* this delivery system is enticing for the treatment of *P. aeruginosa* because there are well known suicidal genetic elements in *P. aeruginosa* that could be used by this system [57].

Phages are able to produce and deliver alginase that can disrupt the capsule formed by these microbes and disturb the biofilm formation [58]. Biofilm formation in cystic fibrosis patients can allow for persistent, antibiotic resistant infections that can be life threatening [59]. In vitro studies have exhibited that bacteriophage have the ability to penetrate biofilms created by *P. aeruginosa*, which makes them an especially attractive delivery system for treatments [60].

While experiments examining the ability of phages to treat *P. aeruginosa* infection in lungs is lacking, there have been several in vitro showing that phages are able to treat infection. A study examining the ability of phages to treat *P. aeruginosa* infection in burn wounds of mice has demonstrated that a single dose of phages was able to significantly decrease the mortality of mice. This reinforces the idea that phages are a possible treatment for infection in patients [61]. Furthermore, a study examining the efficacy of inhaled phages against *Mycobacterium avium* found that mice infected with *M. avium*, then administered phages with *M. smegmatis*, a nonvirulent mycobacterium, experienced a significant decrease of *M. avium* in the spleen [62]. These two studies, while not directly examining the ability of inhaled phages to treat *P. aeruginosa* infection in CF patients, show that phage therapy is both effective against *P. aeruginosa* in vivo and work well when inhaled.

Aerosolized bacteriophage and their utilization as delivery methods for bactericidal proteins and/or enzymes make them enticing for use in treatment of *P. aeruginosa* infections in cystic fibrosis patients. More research is necessary to fully determine if using bacteriophage for treatment in humans is possible.

### Clinical Trials

Although most of the nanoparticles discussed are still being developed, one drug has undergone clinical trials, Arikayce™. Arikayce™ is a liposomal amikacin treatment and has been FDA approved for the treatment of mycobacterium avium complex (MAC) lung disease. There were clinical trials for this drug and its effectiveness for treating *P. aeruginosa* in patients with cystic fibrosis. Although, these are early stages and some research will need to address limitations of this compound in order to increase safety, some preliminary clinical trials have been done (Table 1) [63,64,65,66,67].

## 7. Conclusions

In this review we examined the advancements of drug delivery for the treatment of *P. aeruginosa.* Many antibiotics that are being used to control this infection in patients with CF need to be administered often and in high doses to attain therapeutic concentrations. The major obstacle for drug and therapeutic development for these patients is the thickness of their mucus that makes it hard to penetrate in the lungs and the biofilm of *P. aeruginosa*. Through the use of drug delivery systems utilizing nano-technological approaches such as liposomes and nanoparticles, the effectiveness of antibiotics increases due to the sustained release and targeted delivery at the site of infection. Bacteriophages are being investigated as a novel mechanism to deliver bactericidal proteins or enzymes to combat many different types of infections, including those with *P. aeruginosa*.

It is important to consider that these novel approaches are only at early stages and more research needs to be done in order to move towards the clinical trials phase. Nevertheless, the great advances in the technology and the multidisciplinary approach that is being promotes in this era, are facilitating the discovery of novel alternatives for old infections.

## Figures and Tables

**Table 1:** Summary of clinical trials.

Title of Clinical Trial	Phase	Participants	Location	Begin Date	End Date	Result
Safety/Tolerability Study of Arikayce™ in Cystic Fibrosis Patients With Chronic Infection Due to *P. aeruginosa*	1 and 2	41	USA	January 2008	June 2009	Safe for use
Extension Study of Liposomal Amikacin for Inhalation in Cystic Fibrosis (CF) Patients With Chronic *P. aeruginosa* (Pa) Infection	3	206	Austria, Belgium, Bulgaria, Canada, Denmark, France, Germany, Greece, Hungary, Ireland, Italy, Netherlands, Poland, Serbia, Slovakia, Spain, United Kingdom	5 October 2012	16 July 2015	Had Adverse Events throughout study
Multidose Safety and Tolerability Study of Dose Escalation of Liposomal Amikacin for Inhalation (ARIKACE™)	1 and 2	66	Belgium, Hungary, North Macedonia, Poland, Serbia, Slovakia, Ukraine	22 February 2007	27 February 2008	There were some clinically significant laboratory abnormalities
Study of Dose Escalation of Liposomal Amikacin for Inhalation (ARIKAYCE™)—Extension Phase	2	49	Belgium, Hungary, North Macedonia, Poland, Serbia, Slovakia, Ukraine	8 January 2009	2 November 2010	Adverse events of 560 mg dose of Arikayce administered for six cycles in eighteen months
Study to Evaluate Arikayce™ in CF Patients With Chronic *P. aeruginosa* Infections	3	302	Austria, Belgium, Bulgaria, Canada, Denmark, France, Germany, Greece, Hungary, Ireland, Italy, Netherlands, Poland, Serbia, Slovakia, Spain, Sweden, United Kingdom	29 February 2012	June 2013	Adverse effects

## References

[1] Bethesda C.F.F. 4550 M.A.S. 1100 N.; Md 20814301-951-4422 800-344-4823 about Cystic Fibrosis. http://what-is-cf/about-cystic-fibrosis/.

[2] Cystic Fibrosis—Symptoms and Causes. https://www.mayoclinic.org/diseases-conditions/cystic-fibrosis/symptoms-causes/syc-20353700.

[3] Elborn J.S. (2016). Cystic fibrosis. Lancet.

[4] Pseudomonas. http://textbookofbacteriology.net/pseudomonas_2.html.

[5] Centers for Disease Control and Prevention (US) (2019). Antibiotic Resistance Threats in the United States.

[6] Marshall B. (2016). Highlights of the 2014 Patient Registry.

[7] Ciofu O., Rojo-Molinero E., Macià M.D., Oliver A. (2017). Antibiotic treatment of biofilm infections. APMIS.

[8] Mihai M.M., Giurcaneanu C., Popa L.G., Nitipir C., Popa M.I. (2015). Controversies and challenges of chronic wound infection diagnosis and treatment. Mod. Med..

[9] Mihai M.M., Dima M.B., Dima B., Holban A.M. (2019). Nanomaterials for Wound Healing and Infection Control. Materials.

[10] Ciobanu S., Mihai M.M., Popa L.G., Giurcaneanu C., Popa M.I. (2015). Considerations on the pathogenesis of chronic venous ulcers—Review/Consideratii asupra patogenezei ulcerelor venoase cronice—Review. Infectio ro.

[11] Grassi L., Di Luca M., Maisetta G., Rinaldi A.C., Esin S., Trampuz A., Batoni G. (2017). Generation of Persister Cells of Pseudomonas aeruginosa and Staphylococcus aureus by Chemical Treatment and Evaluation of Their Susceptibility to Membrane-Targeting Agents. Front. Microbiol..

[12] Ong H.X., Traini D., Cipolla D., Gonda I., Bebawy M., Agus H., Young P.M. (2012). Liposomal Nanoparticles Control the Uptake of Ciprofloxacin Across Respiratory Epithelia. Pharm. Res..

[13] Pseudomonas Aeruginosa. http://www.antimicrobe.org/b112.asp.

[14] Podgoreanu P., Negrea S.M., Buia R., Delcaru C., Trusca S.B., Lazar V., Chifiriuc M.C. (2019). Alternative strategies for fighting multidrug resistant bacterial infections. Biointerface Res. Appl. Chem..

[15] Soto-Chilaca G.A., Mejia-Garibay B., Navarro-Amador R., Ramirez-Corona N., Palou E., Lopez-Malo A. (2019). Cinnamaldehyde-loaded chitosan nanoparticles: Characterization and antimicrobial activity. Biointerface Res. Appl. Chem..

[16] Pompilio A., Geminiani C., Bosco D., Rana R., Aceto A., Bucciarelli T., Scotti L., Di Bonaventura G. (2018). Electrochemically Synthesized Silver Nanoparticles Are Active Against Planktonic and Biofilm Cells of Pseudomonas aeruginosa and Other Cystic Fibrosis-Associated Bacterial Pathogens. Front. Microbiol..

[17] Liao S., Zhang Y., Pan X., Zhu F., Jiang C., Liu Q., Cheng Z., Dai G., Wu G., Wang L. (2019). Antibacterial activity and mechanism of silver nanoparticles against multidrug-resistant Pseudomonas aeruginosa. Int. J. Nanomed..

[18] Chen Q., Shah K.N., Zhang F., Salazar A.J., Shah P.N., Li R., Sacchettini J.C., Wooley K.L., Cannon C.L. (2019). Minocycline and Silver Dual-Loaded Polyphosphoester-Based Nanoparticles for Treatment of Resistant Pseudomonas aeruginosa. Mol. Pharm..

[19] Grumezescu A.M., Gestal M.C., Holban A.M., Grumezescu V., Vasile B.Ș., Mogoantă L., Iordache F., Bleotu C., Mogoșanu G.D. (2014). Biocompatible Fe3O4 Increases the Efficacy of Amoxicillin Delivery against Gram-Positive and Gram-Negative Bacteria. Molecules.

[20] Grumezescu A.M., Andronescu E., Holban A.M., Ficai A., Ficai D., Voicu G., Grumezescu V., Balaure P.C., Chifiriuc C.M. (2013). Water dispersible cross-linked magnetic chitosan beads for increasing the antimicrobial efficiency of aminoglycoside antibiotics. Int. J. Pharm..

[21] Grumezescu V., Holban A., Iordache F., Socol G., Mogoşanu G., Grumezescu A., Ficai A., Vasile B., Chifiriuc M., Maniu H. (2014). MAPLE fabricated magnetite@eugenol and (3-hidroxybutyric acid-co-3-hidroxyvaleric acid)—Polyvinyl alcohol microspheres coated surfaces with anti-microbial properties. Appl. Surf. Sci..

[22] Grumezescu A.M., Andronescu E., Ficai A., Bleotu C., Mihaiescu D.E., Chifiriuc M.C. (2012). Synthesis, characterization and in vitro assessment of the magnetic chitosan–carboxymethylcellulose biocomposite interactions with the prokaryotic and eukaryotic cells. Int. J. Pharm..

[23] Marková Z., Siskova K., Filip J., Safarova K., Prucek R., Panacek A., Kolář M., Zboril R. (2012). Chitosan-based synthesis of magnetically-driven nanocomposites with biogenic magnetite core, controlled silver size, and high antimicrobial activity. Green Chem..

[24] Omri A., Suntres Z.E., Shek P.N. (2002). Enhanced activity of liposomal polymyxin B against Pseudomonas aeruginosa in a rat model of lung infection. Biochem. Pharmacol..

[25] Wong J.P., Yang H., Blasetti K.L., Schnell G., Conley J., Schofield L.N. (2003). Liposome delivery of ciprofloxacin against intracellular Francisella tularensis infection. J. Control. Release.

[26] Saari S.M., Vidgren M.T., Herrala J., Turjanmaa V.M.H., Koskinen M.O., Nieminen M.M. (2002). Possibilities of formoterol to enhance the peripheral lung deposition of the inhaled liposome corticosteroids. Respir. Med..

[27] Weers J., Metzheiser B., Taylor G., Warren S., Meers P., Perkins W.R. (2009). A gamma scintigraphy study to investigate lung deposition and clearance of inhaled amikacin-loaded liposomes in healthy male volunteers. J. Aerosol Med. Pulm. Drug Deliv..

[28] Okusanya Ó.O., Bhavnani S.M., Hammel J., Minic P., Dupont L.J., Forrest A., Mulder G.-J., Mackinson C., Ambrose P.G., Gupta R. (2009). Pharmacokinetic and Pharmacodynamic Evaluation of Liposomal Amikacin for Inhalation in Cystic Fibrosis Patients with Chronic Pseudomonal Infection. Antimicrob. Agents Chemother..

[29] Allen S.D., Sorensen K.N., Neial M.J., Durrant C., Proffit R.T. (1994). Prophylactic efficacy of aerosolized liposomal (AmBisome) and non-lipsomal (Fungizone) amphotericin B in murine pulmonary aspergillosis. J. Antimicrob. Chemother..

[30] Slobbe L., Boersma E., Rijnders B.J.A. (2008). Tolerability of prophylactic aerosolized liposomal amphotericin-B and impact on pulmonary function: Data from a randomized placebo-controlled trial. Pulm. Pharmacol. Ther..

[31] Vij N., Min T., Marasigan R., Belcher C.N., Mazur S., Ding H., Yong K.-T., Roy I. (2010). Development of PEGylated PLGA nanoparticle for controlled and sustained drug delivery in cystic fibrosis. J. Nanobiotechnol..

[32] Bruinenberg P., Blanchard J.D., Cipolla D.C., Dayton F., Mudumba S., Gonda I. (2010). Inhaled Liposomal Ciprofloxacin: Once a Day Management of Respiratory Infections. Respir. Drug Deliv..

[33] Meers P., Neville M., Malinin V., Scotto A.W., Sardaryan G., Kurumunda R., Mackinson C., James G., Fisher S., Perkins W.R. (2008). Biofilm penetration, triggered release and in vivo activity of inhaled liposomal amikacin in chronic Pseudomonas aeruginosa lung infections. J. Antimicrob. Chemother..

[34] Omri A., Beaulac C., Bouhajib M., Montplaisir S., Sharkawi M., Lagace J. (1994). Pulmonary retention of free and liposome-encapsulated tobramycin after intratracheal administration in uninfected rats and rats infected with Pseudomonas aeruginosa. Antimicrob. Agents Chemother..

[35] Beaulac C., Clément-Major S., Hawari J., Lagacé J. (1996). Eradication of mucoid Pseudomonas aeruginosa with fluid liposome-encapsulated tobramycin in an animal model of chronic pulmonary infection. Antimicrob. Agents Chemother..

[36] Bakker-Woudenberg I.A.J.M., ten Kate M.T., Guo L., Working P., Mouton J.W. (2002). Ciprofloxacin in Polyethylene Glycol-Coated Liposomes: Efficacy in Rat Models of Acute or Chronic Pseudomonas aeruginosa Infection. Antimicrob. Agents Chemother..

[37] Ghaffari S., Varshosaz J., Saadat A., Atyabi F. (2010). Stability and antimicrobial effect of amikacin-loaded solid lipid nanoparticles. Int. J. Nanomed..

[38] Nafee N., Husari A., Maurer C.K., Lu C., de Rossi C., Steinbach A., Hartmann R.W., Lehr C.-M., Schneider M. (2014). Antibiotic-free nanotherapeutics: Ultra-small, mucus-penetrating solid lipid nanoparticles enhance the pulmonary delivery and anti-virulence efficacy of novel quorum sensing inhibitors. J. Control Release.

[39] Varshosaz J., Ghaffari S., Mirshojaei S.F., Jafarian A., Atyabi F., Kobarfard F., Azarmi S. (2013). Biodistribution of amikacin solid lipid nanoparticles after pulmonary delivery. BioMed Res. Int..

[40] Cavalli R., Zara G.P., Caputo O., Bargoni A., Fundarò A., Gasco M.R. (2000). Transmucosal transport of tobramycin incorporated in SLN after duodenal administration to rats. Part I—A pharmacokinetic study. Pharmacol. Res..

[41] Bargoni A., Cavalli R., Zara G.P., Fundarò A., Caputo O., Gasco M.R. (2001). Transmucosal transport of tobramycin incorporated in solid lipid nanoparticles (sln) after duodenal administration to rats. Part II—Tissue distribution. Pharmacol. Res..

[42] Pastor M., Moreno-Sastre M., Esquisabel A., Sans E., Viñas M., Bachiller D., Asensio V.J., Pozo Á.D., Gainza E., Pedraz J.L. (2014). Sodium colistimethate loaded lipid nanocarriers for the treatment of Pseudomonas aeruginosa infections associated with cystic fibrosis. Int. J. Pharm..

[43] Ryan G.M., Kaminskas L.M., Kelly B.D., Owen D.J., McIntosh M.P., Porter C.J.H. (2013). Pulmonary Administration of PEGylated Polylysine Dendrimers: Absorption from the Lung versus Retention within the Lung Is Highly Size-Dependent. Mol. Pharm..

[44] Ungaro F., d’Angelo I., Coletta C., d’Emmanuele di Villa Bianca R., Sorrentino R., Perfetto B., Tufano M.A., Miro A., La Rotonda M.I., Quaglia F. (2012). Dry powders based on PLGA nanoparticles for pulmonary delivery of antibiotics: Modulation of encapsulation efficiency, release rate and lung deposition pattern by hydrophilic polymers. J. Control Release.

[45] Günday Türeli N., Torge A., Juntke J., Schwarz B.C., Schneider-Daum N., Türeli A.E., Lehr C.-M., Schneider M. (2017). Ciprofloxacin-loaded PLGA nanoparticles against cystic fibrosis P. aeruginosa lung infections. Eur. J. Pharm. Biopharm..

[46] Hua X., Tan S., Bandara H.M.H.N., Fu Y., Liu S., Smyth H.D.C. (2014). Externally Controlled Triggered-Release of Drug from PLGA Micro and Nanoparticles. PLoS ONE.

[47] Cheow W.S., Hadinoto K. (2012). Lipid-polymer hybrid nanoparticles with rhamnolipid-triggered release capabilities as anti-biofilm drug delivery vehicles. Particuology.

[48] Abdelghany S.M., Quinn D.J., Ingram R.J., Gilmore B.F., Donnelly R.F., Taggart C.C., Scott C.J. (2012). Gentamicin-loaded nanoparticles show improved antimicrobial effects towards Pseudomonas aeruginosa infection. Int. J. Nanomed..

[49] Deacon J., Abdelghany S.M., Quinn D.J., Schmid D., Megaw J., Donnelly R.F., Jones D.S., Kissenpfennig A., Elborn J.S., Gilmore B.F. (2015). Antimicrobial efficacy of tobramycin polymeric nanoparticles for Pseudomonas aeruginosa infections in cystic fibrosis: Formulation, characterisation and functionalisation with dornase alfa (DNase). J. Control Release.

[50] D’Angelo I., Casciaro B., Miro A., Quaglia F., Mangoni M.L., Ungaro F. (2015). Overcoming barriers in Pseudomonas aeruginosa lung infections: Engineered nanoparticles for local delivery of a cationic antimicrobial peptide. Colloids Surf. B Biointerfaces.

[51] Flockton T.R., Schnorbus L., Araujo A., Adams J., Hammel M., Perez L.J. (2019). Inhibition of Pseudomonas aeruginosa Biofilm Formation with Surface Modified Polymeric Nanoparticles. Pathogens.

[52] Ho K. (2001). Bacteriophage Therapy for Bacterial Infections: Rekindling a Memory from the Pre-Antibiotics Era. Perspect. Biol. Med..

[53] Golshahi L., Lynch K.H., Dennis J.J., Finlay W.H. (2011). In vitro lung delivery of bacteriophages KS4-M and ΦKZ using dry powder inhalers for treatment of Burkholderia cepacia complex and Pseudomonas aeruginosa infections in cystic fibrosis. J. Appl. Microbiol..

[54] Matinkhoo S., Lynch K.H., Dennis J.J., Finlay W.H., Vehring R. (2011). Spray-dried Respirable Powders Containing Bacteriophages for the Treatment of Pulmonary Infections. J. Pharm. Sci..

[55] Sahota J.S., Smith C.M., Radhakrishnan P., Winstanley C., Goderdzishvili M., Chanishvili N., Kadioglu A., O’Callaghan C., Clokie M.R.J. (2015). Bacteriophage Delivery by Nebulization and Efficacy Against Phenotypically Diverse Pseudomonas aeruginosa from Cystic Fibrosis Patients. J. Aerosol Med. Pulm. Drug Deliv..

[56] Westwater C., Kasman L.M., Schofield D.A., Werner P.A., Dolan J.W., Schmidt M.G., Norris J.S. (2003). Use of Genetically Engineered Phage To Deliver Antimicrobial Agents to Bacteria: An Alternative Therapy for Treatment of Bacterial Infections. Antimicrob. Agents Chemother..

[57] Molin S., Jensen L.B., Kristensen C.S., Givskov M., Ramos J.L., Bej A.K. (1993). Suicidal Genetic Elements and Their Use In Biological Containment Of Bacteria. Annu. Rev. Microb..

[58] Glonti T., Chanishvili N., Taylor P.W. (2010). Bacteriophage-derived enzyme that depolymerizes the alginic acid capsule associated with cystic fibrosis isolates of Pseudomonas aeruginosa. J. Appl. Microbiol..

[59] Høiby N., Ciofu O., Bjarnsholt T. (2010). Pseudomonas aeruginosa biofilms in cystic fibrosis. Future Microbiol..

[60] Sharma G., Rao S., Bansal A., Dang S., Gupta S., Gabrani R. (2014). Pseudomonas aeruginosa biofilm: Potential therapeutic targets. Biologicals.

[61] McVay C.S., Velásquez M., Fralick J.A. (2007). Phage Therapy of Pseudomonas aeruginosa Infection in a Mouse Burn Wound Model. Antimicrob. Agents Chemother..

[62] Danelishvili L., Young L.S., Bermudez L.E. (2006). In Vivo Efficacy of Phage Therapy for Mycobacterium avium Infection As Delivered by a Nonvirulent Mycobacterium. Microb. Drug Resist..

[63] Safety/Tolerability Study of Arikayce^TM^ in Cystic Fibrosis Patients with Chronic Infection Due to Pseudomonas Aeruginosa—Full Text View—ClinicalTrials.gov. https://clinicaltrials.gov/ct2/show/NCT00558844.

[64] Extension Study of Liposomal Amikacin for Inhalation in Cystic Fibrosis (CF) Patients with Chronic Pseudomonas Aeruginosa (Pa) Infection—Full Text View—ClinicalTrials.gov. https://clinicaltrials.gov/ct2/show/NCT01316276.

[65] Multidose Safety and Tolerability Study of Dose Escalation of Liposomal Amikacin for Inhalation (ARIKACE^TM^)—Full Text View—ClinicalTrials.gov. https://clinicaltrials.gov/ct2/show/NCT00777296.

[66] Study of Dose Escalation of Liposomal Amikacin for Inhalation (ARIKAYCE^TM^)—Extension Phase—Full Text View—ClinicalTrials.gov. https://clinicaltrials.gov/ct2/show/NCT03905642.

[67] Study to Evaluate Arikayce^TM^ in CF Patients With Chronic Pseudomonas Aeruginosa Infections—Full Text View—ClinicalTrials.gov. https://clinicaltrials.gov/ct2/show/NCT01315678.

